# Joint Consideration of LDL-C and Polygenic Risk for Coronary Heart Disease Risk Assessment

**DOI:** 10.1016/j.jacadv.2025.102228

**Published:** 2025-10-08

**Authors:** Carlos Iribarren, Meng Lu, Roberto Elosua, Martha Gulati, Nathan D. Wong, Jamal S. Rana

**Affiliations:** a Kaiser Permanente Northern California Division of Research, Pleasanton, California, USA; b Cardiovascular Epidemiology and Genetics, Institut Hospital del Mar d'Investigacions Mèdiques (IMIM), Barcelona, Spain; c Spain and CIBER Cardiovascular Diseases (CIBERCV), Barcelona, Spain; d Faculty of Medicine, University of Vic-Central University of Catalonia (UVic-UCC), Vic, Spain; e Barbra Streisand Women’s Heart Center, Smidt Heart Institute, Cedars-Sinai Medical Center, Los Angeles, California, USA; f Heart Disease Prevention Program, Mary and Steve Wen Cardiovascular Division, Department of Medicine, University of California Irvine, Irvine, California, USA; g Department of Cardiology, The Permanente Medical Group, Kaiser Permanente Oakland Medical Center, Oakland, California, USA

**Keywords:** genetics, LDL-cholesterol, polygenic risk score

**Figure:**
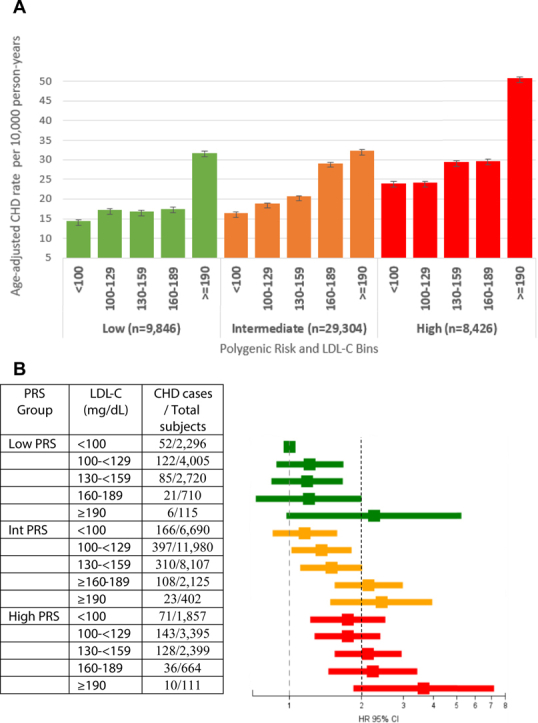

Elevated low-density lipoprotein-cholesterol (LDL-C) is a major modifiable risk factor for coronary heart disease (CHD).^1^^,^^2^ A body of evidence indicates that polygenic risk scores (PRS) confer an independent risk of CHD that is present from birth.^3^

**What is the clinical question being addressed?**

Is CHD risk associated with LDL-C amplified by PRS?

**What is the main finding?**

Among statin-naive individuals with no diabetes, the threshold of increased risk varied by PRS risk group. These findings have implications for primary prevention for CHD.

A recent UK Biobank study suggests that the CHD risk imparted by LDL-C may be modulated by polygenic risk.^4^ In this study, individuals with high PRS and LDL-C 130 to <160 mg/dL showed comparable increased CHD risk (HR: 2.2) to those with intermediate PRS and LDL ≥190 mg/dL (HR: 2.3). Furthermore, CHD risk in individuals with low PRS did not follow the stepwise increase observed in the other PRS groups. Our aim was to examine whether the CHD risk associated with high LDL-C levels is amplified by background polygenic risk in a large, multiethnic real-world cohort in Northern California, USA, using a commercially available CHD PRS.

This study made use of genome-wide genetic data obtained from the Genetic Epidemiology Research on Aging cohort of adult members of Kaiser Permanente of Northern California. Recruitment, data collection, genotyping, and outcome ascertainment through December 31, 2022, have been published.^5^ The study was approved by the Kaiser Foundation Research Institute Institutional Review Board, and all individuals provided informed consent.

The initial Genetic Epidemiology Research on Aging cohort comprised 110,226 individuals. Sequential baseline exclusions included incomplete genetic data for estimation of the PRS and/or principal components of genetic ancestry (n = 12,293), age <30 or >79 years (n = 8,389), prevalent CHD (n = 2,600), on statins (28,399), missing LDL-C (n = 6,218), missing covariates (body mass index [BMI], smoking status, diabetes, hypertension; n = 1,781), and diabetes (n = 3,010). We excluded those with diabetes because it was considered a “CHD-equivalent” in the 2013 American College of Cardiology/American Heart Association cholesterol treatment guideline update and most patients with diabetes in our health plan are prescribed statins at some point, distorting the prospective association of LDL-C with CHD. The final cohort comprised 47,576 statin-naive individuals free of diabetes. After a mean (SD) follow-up time of 13.8 (3.8) years, 1,678 incident CHD events (including myocardial infarction, coronary revascularization procedures, angina pectoris, or CHD death) occurred. Further details of the PRS (CARDIO inCode-Score CHD PRS, GENinCode Plc) description and validation can be found elsewhere.^5^ LDL-C was estimated using the Friedewald equation (or measured with the direct method when triglycerides exceeded 400 mg/dL) at a Clinical Laboratory Improvement Amendments-certified KP Regional Laboratory (Berkeley, California, USA).

We estimated age-adjusted rates per 10,000 person-years of incident CHD by joint categories of PRS and LDL-C including LDL-C (in mg/dL) <100, 100 to 129, 130 to 159, 160 to 189, and ≥190 in each of the PRS groups (low, first quintile; intermediate, quintiles 2-4; high, fifth quintile) using Poisson regression. We then performed Cox proportional hazards regression with the main exposure being joint categories of PRS and LDL-C groups, with low PRS and LDL-C <100 mg/dL as the reference group, adjusting for age, sex, 5 principal components of genetic ancestry, BMI, smoking, and hypertension. In separate fully-adjusted Cox models, we tested for interaction between continuous LDL-C and PRS and for categorical LDL-C and PRS. Furthermore, we performed restricted cubic spline regression analysis stratifying by polygenic risk.

At baseline, the cohort had a mean (SD) age of 58 (10) years, and 62% of the participants were female. Approximately 82% self-identified as European, 3% as African-American, 7% as Latino, and 8% as Asian. About 5% were current smokers; 34% former smokers. About 18% had a BMI in the obesity range and 36% had hypertension. Mean (SD) LDL-C was 121 (29) mg/dL; mean (SD) high-density lipoprotein-cholesterol was 58 (16) mg/dL.

In the low polygenic risk group, CHD rates remain low until LDL-C reaches 190 mg/dL (Figure 1A). In the intermediate polygenic risk group, CHD rates increase steadily with increasing LDL-C, with a threshold at 160 mg/dL. In the high polygenic risk group, we see increased CHD rates even at a threshold of LDL-C of 100 mg/dL (HR for those below 100 mg/dL: 1.75; 95% CI: 1.22-2.40; *P* < 0.0001) and above. The restricted cubic spline regression analysis stratifying by polygenic risk showed consistent results (data not shown). The highest CHD rate was observed for the combination of both high polygenic risk and severe hypercholesterolemia.

**Figure 1:**
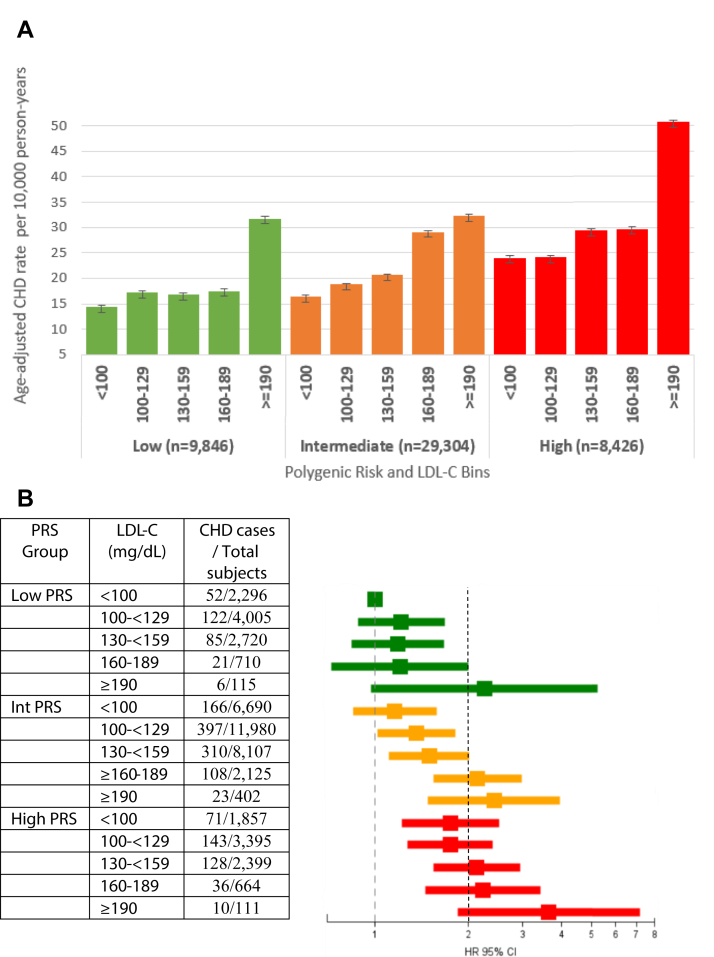
Age-Adjusted CHD Rates per 10,000 Person-Years by Polygenic Risk and LDL-C Bins and the Number of CHD Events, Subjects at Risk and Forest Plot of Adjusted HR (95% CI) (A) Age-adjusted CHD rates per 10,000 person-years by polygenic risk and LDL-C bins. (B) Number of CHD events, subjects at risk and forest plot of adjusted HR (95% CI). CHD = coronary heart disease; PRS = polygenic risk scores.

There were nonstatistically significant associations between LDL-C and incident CHD among low polygenic risk individuals, although those with severe hypercholesterolemia (≥190 mg/dL) had a markedly elevated HR (2.3; *P* = 0.06) (Figure 1B). In the intermediate polygenic risk group, there was a steady increased risk of CHD as LDL-C increased, with HR >2.0 starting at 160 mg/dL. In the high polygenic risk group, HRs of CHD began to increase markedly starting at LDL-C of 100 mg/dL and the combination of both high polygenic risk and LDL-C >190 mg/dL was associated with a HR of 3.6. We found no statistically significant interaction between continuous LDL-C and PRS (*P* = 0.34) or between categorical LDL-C and PRS (*P* = 0.60).

In agreement with Bolli et al among White participants in the UK Biobank,^4^ the effect of LDL-C on CHD risk was modulated by polygenic risk such that the LDL-C threshold of increased risk was different depending on the PRS group. However, we saw a signal for severe hypercholesterolemia in the low PRS group, albeit it was only marginally significant (driven by 6 events in 115 people). This is consistent with lack of LDL-C*PRS interaction in our data. Also, unlike Bolli et al, the adjusted risk of high polygenic risk and LDL 130 to 160 mg/dL (HR: 2.1) was commensurate with intermediate polygenic risk and LDL 160 to 190 mg/dL (HR: 2.1); whereas in their study, it was commensurate with intermediate polygenic risk and LDL ≥190 mg/dL. Differences in settings, populations, and methodological approaches may explain these differences.

We recognize limitations. First, our cohort participants were all members of Kaiser Permanente of Northern California, therefore findings may not fully generalize to uninsured populations. Second, the majority of patients were of European descent. Third, we did not have genetic assessment of familial hypercholesterolemia (FH) and is possible that the increased risk of those with LDL-C of 190 mg/dL or higher may have been driven by FH genes.

In summary, polygenic risk modulated the association of LDL-C with incident CHD such that there was significantly increased risk even at LDL-C 100 mg/dL and above among individuals with high polygenic risk. Furthermore, we found a remarkably increased risk (HR: >3.5) with the combination of high polygenic risk and LDL ≥190 mg/dL, which is in the order of magnitude of the risk reported for FH. These results have important clinical implications for lipid management in asymptomatic populations and support more aggressive management and primary prevention for individuals with high polygenic risk.

## Funding support and author disclosures

This study was funded by a grant from GEN inCODE, PLC. Dr Iribarren has received a research grant from GENinCode Plc. Dr Elosua is a member of the scientific advisory board of GENinCode Plc and inventor of a patent based on the CARDIO inCode-Score CHD Polygenic Risk Score (PRS) granted to and owned by GENinCode Plc. Dr. Wong is a consultant to Amgen, Novartis, and Ionis. All other authors have reported that they have no relationships relevant to the contents of this paper to disclose.
